# Hormonal and molecular characterization of calcium oxalate stone formers predicting occurrence and recurrence

**DOI:** 10.1007/s00240-023-01440-8

**Published:** 2023-04-24

**Authors:** Ahmed M. Elshal, Heba Shamshoun, Amira Awadalla, Ramy Elbaz, Asmaa E. Ahmed, Omali Y. El-khawaga, Ahmed A. Shokeir

**Affiliations:** 1 Department of Urology, Urology and Nephrology Center, Mansoura University, Mansoura, Egypt; 2 Center of Excellence for Genome and Cancer Research, Urology and Nephrology Center, Mansoura University, Mansoura, Egypt; 3 Faculty of Science, Mansoura University, Mansoura, Egypt

**Keywords:** Calcium oxalate, Androgen, Urolithiasis, Stone

## Abstract

The purpose of the study is to investigate the role of sex hormones, androgen receptors (ARs) and miRNA/CSF-1 in occurrence and recurrence of calcium oxalate (CaOx) renal urolithiasis. In this prospective study, 74 patients with CaOx stones; stone formers group (SFG) and 40 healthy subjects; control group were compared. SFG includes both de novo and recurrent cases. Steroid sex hormone plasma assay including testosterone, free testosterone, dihydrotestosterone, estradiol, and sex hormone binding globulin was analyzed. ARs, miRNA-185-5p and CSF-1 expression were compared between the groups. SFG showed significant higher ARs and miRNA-185-5p expression (3.7 ± 1.3, 1.8 ± 0.4, respectively) than control group (1 ± 0.08 and 1 ± 0.07, respectively) (*p* < 0.05). However, CSF-1 expression was significantly lower in stone formers than control group (0.4 ± 0.19 vs 1 ± 0.1, respectively) (*p* < 0.05). No differences were detected between de novo and recurrent SFG regarding sex hormones, AR, miRNA or CSF-1 expression. Our data suggest the important role of AR, miRNA and CSF-1 signaling in human nephrolithiasis pathogenesis.

## Introduction

Urolithiasis represents one of the most common urologic health care problems worldwide. Estimates of calcium oxalate (CaOx)/calcium phosphate (CaPh) stones occurrence are as high as 80% of all renal stones. The underlying pathophysiology of the calcium-based renal stones is complex and multifactorial with most of these stones which occur in healthy individuals because of metabolic derangements which involve an imbalance between stone formers and stone inhibitors. The most common of these derangements are idiopathic hypercalciuria, hyperoxaluria and hypocitraturia [1].

CaOx stones formation can be affected by infiltrating macrophages which include pro-inflammatory macrophages or M1 promoting crystals deposition and anti-inflammatory or M2 macrophages phagocytosing ca-oxalate stone formation directly [2].

Regarding the age and sex prevalence of renal stones, it is more common in males compared to females by about 2–3:1. This prevalence gap is mainly present in reproductive age between 15 and 49 years and lowest in postmenopausal females with low estrogen which propose that estrogen can serve as a protective hormone against urolithiasis [3, 4]. However, recent evidence suggests that the difference in incidence between men and women is narrowing. Using National Health and Nutrition Examination Survey (NHANES) data, Stamatelou et al. reported a slight decrease in the male-to-female ratio of stone disease, from 1.75 (between 1976 and 1980) to 1.54 (between 1988 and 1994) [5]. Recently, NHANES data (2007–2010) showed a stone prevalence of 10.6% in men and 7.1% in women for a ratio of 1.49:1 [6]. Lieske et al. found a peak incidence in the age group 60 to 69 years in men with just a slightly higher incidence in women in the age groups 30 to 39 and 60 to 69 [7].

In addition, androgen-deprivation therapy (ADT) use in patients with prostate cancer results in approximately one-third lower risk of subsequent renal calculi [8] which may suggest a promoting effect of male androgens on renal stones formation. This effect has been established experimentally in rats in some trials [9].

Testosterone appears to promote stone formation by suppressing osteopontin expression in the kidneys and increasing urinary oxalate excretion, and this action has been found that is reversed with estrogen [10]. Another possible mechanism for promoting role of testosterone on calcium oxalate stone formation may be due to increase in urinary uric acid excretion [3] and increase in hepatic synthesis of glycolic acid oxidase (GAO), an enzyme essentially involved in primary hyperoxaluria [11].

This action of sex hormones could be done either through alternation of serum hormone levels or expression of their receptors. It was found that androgen receptors (AR) stimulation increases miRNA-185-5p which inhibit macrophages colony-stimulating factor 1 (CSF-1) result in inhibition of M2-mediated CaOx crystals phagocytosis [12–14]. However, this action was demonstrated only using human cell lines via in vitro studies and rat models. Furthermore, higher testosterone levels were reported in renal stones patients. Considering the role of estrogen, data are sparse due to lack of assessment of the role of estrogen or absence of depictable inhibitory role of estrogen on stones formation [12–14]. In addition, the relation between sex hormones and calcium oxalates stones recurrence was not assessed before in any trial.

Until now, there is a shortage of data regarding the association between sex hormones and the prevalence of urolithiasis and the utility of this association in clinical practice. This study was conducted looking for an association between sex hormones and calcium oxalate stone formation. Moreover, the relation between sex hormones and recurrence of calcium oxalate stones was assessed.

## Patients and methods

- Study design and groups

This is a prospective controlled study that was performed between June 2020 and June 2021 after approval of the local ethical committee.

Patients who were diagnosed with calcium oxalate (CaOx) stones on further stone analysis either as pure CaOx stones or mixed stones were allocated as stone formers group (SFG) and compared against healthy volunteers who were selected from same age range with no history or radiological evidence of urinary stones and considered as “control” group.

Stone formers group was subdivided into: “denovo” group included patients who presented with renal stones for the first time, and they were compared against “recurrent” group who presented with previous episodes of renal stones that necessitated active intervention.

- Inclusion and exclusion criteria

Inclusion criteria included male patients between 18 and 60 years who were diagnosed with renal stones and were admitted to our center for active stone intervention. Eligible subjects were asked to sign an informed consent.

Exclusion criteria included patients with known hyperthyroidism, hyperparathyroidism, recurrent urinary tract infections, renal and liver diseases, intestinal malabsorption, or metabolic syndromes were excluded from the study. In addition, patients with known structural or functional abnormality of the urinary tract such as vesico-ureteric reflux, neuropathic bladder or pelvi-uretral junction obstruction were also excluded from the study.

- Intervention

All patients who were diagnosed with renal stones were managed with standard percutaneous nephrolithotomy (PNL) or retrograde intrarenal surgery (RIRS) depending on stone volume, location, and surgeons’ preference. Retrieved stones were submitted for stone analysis and calcium oxalate (CaOx) stone formers were included.

- Study workup

All clinical data including history, clinical examination and routine laboratory investigation were collected. The sonographic and computerized tomographic evaluations of the kidneys and urinary tract systems and the diagnosis of renal stone were performed by the attending expert radiologists.

Sample collection for laboratory and molecular analysis was done as following:

24-h urine samples collection for determination the levels of calcium, uric acid, citrate and oxalate.

Stone samples for analysis using FT-IR (Fourier Transform- Infrared) spectroscopy looking for type of stone.

Blood samples were taken for evaluation the level of sex hormones and gene expression.

### Hormone analysis

For hormone analysis, all samples of blood were collected, and each sample was centrifuged at 3000 g for 15 min and the separated plasma then fractionated and stored at − 20 ℃ until hormone assay. Hormones in the plasma samples including testosterone (T), free testosterone (FT), dihydrotestosterone (DHT), estradiol (E2), and sex hormone binding globulin (SHBG) were analyzed by ELISA.

### Gene expression of AR and CSF-1 using real-time PCR

AR mRNA levels were assessed by quantitative RT-qPCR method. Total RNA (0.5 μg) was isolated from the blood samples, and reverse transcribed. Gene expression for Primers for the AR and CSF-1 and human glyceraldehyde-3-phosphate dehydrogenase (GAPDH) were examined by real-time qPCR, using AR primers (Applied Biosystems, Foster City, CA, USA). Gene expressions all data were normalized to GAPDH; expression of one tumor was assumed to be 1 and used as reference. The 2−ΔΔCT method was used to calculate relative amounts of target genes.

### Gene expression of miRNA-185-5p

miRNA technology ingeniously integrates a universal tailing and reverse transcription reaction specific for mature miRNA combined with state-of-the-art primer-assay design technology to enable the accurate expression level measurement of miR-2909.miRNA was extracted from plasma samples using an miRneasy Mini Kit. cDNA was synthesized from 1 μg of total miRNA. Amplification and detection were performed using real-time PCR (step one plus). AR, CSF-1 and miRNA-185-5p

- Study outcomes

The primary outcome was to assess the association between sex hormones and occurrence of calcium oxalate renal stones, while the secondary outcome was to assess the association between these hormones and recurrence of calcium oxalate stones over time.

- Statistical analysis

Continuous variables were presented as mean ± SD for normally distributed and as median and range for non-normally distributed variables. Ordinal and nominal variables were presented as frequency and percentage. Comparison between study and control groups was performed by Student’s *t* test for normally distributed continuous variables and by Mann–Whitney test for asymmetric continuous variables. Chi square and Fisher exact tests was used for comparison between categorical variables. Multivariable analysis by logistic regression was done as appropriate. A *P* value < 0.05 was considered significant. A software SPSS was used for storage and analysis of data.

## Results

### Demographic and perioperative data

Out of 91 male patient who were diagnosed with renal stones and underwent endoscopic treatment with PNL or RIRS between June 2020 and June 2021, 74 patients were diagnosed as CaOx stones and included in the study. Then, they were compared against 40 healthy subjects.

**Fig. 1:**
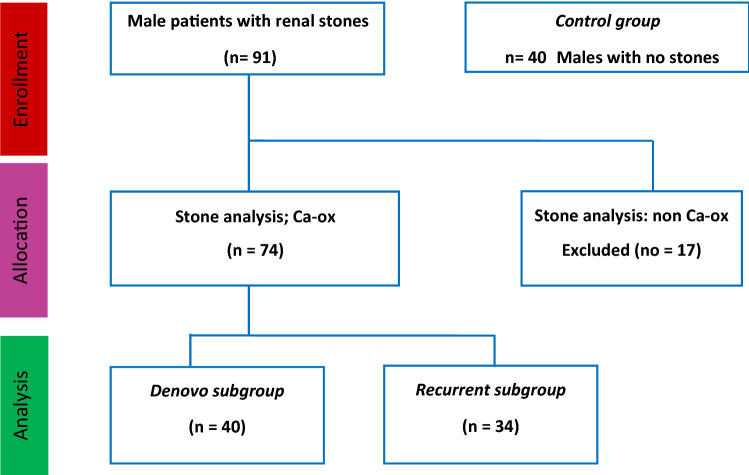
CONSORT flow chart

After that, the 2 subgroups of SFG were compared against each other: 40 patients of de novo group and 34 patients of recurrent group as shown in study’s flow chart (Fig. 1).

The mean ± SD age and BMI in SFG and control groups were (52 ± 14 and 54 ± 10, *P* 0.4) and (30.6 ± 7.3 and 29 ± 5.3, *P* 0.09), respectively. In addition, patients̓ demographics and stone characteristics of de novo and recurrent stone formers group were comparable with no significant difference as shown in Table 1.

**Table 1:** Baseline demographic characteristics of de novo and recurrent stone formers groups

Variable	De novo group	Recurrent group	*P* value
	*N*:40	*N*:34	
Patients’ characteristics			
Age (years)			
Mean ± SD	51 ± 10	53 ± 8	0.3*
BMI (kg/m^2^)			
Mean ± SD	30.1 ± 5.3	28 ± 8.3	0.07*
DM			
NO (%)	4 (10%)	7 (20.6)	0.3^#^
Urinary tract characteristics			
Urine PH			
Mean ± SD	5.7 ± 0.7	5.4 ± 0.7	0.02*
S Cr			
Median (range)	0.9 (0.6–4)	1 (0.4–3)	0.1**
Stone characteristics			
Stone density			
Mean ± SD	1015 ± 306	1021 ± 266	0.9*
Stone diameter			
Median (range)	13 (4–30)	14 (1–40)	0.7**
Stone length			
Median (range)	16 (2–40)	15 (1.6–42)	0.7**
Stone side			
NO (%)			0.6^#^
RT	15 (37.5)	11 (32.4)	
LT	21 (52.5)	17 (50)	0.6^#^
Bilateral	4 (10)	6 (17.6)	

*S Cr* serum creatinine, *BMI* body mass index

*Independent sample *t* test

**Mann–Whitney *U* test

^#^Chi square test

### The primary outcome

As shown in Table 2, the mean ± SD levels of RQ-AR, miRNA-185 and serum estradiol were significantly higher in SFG 3.7 ± 1.3, 1.8 ± 0.4 and 69.5 (20–300), respectively, compared to control group 1 ± 0.08, 1 ± 0.07 and 34 (20–55), respectively, with *p* < 0.001.

**Table 2:** Comparison of hormonal and molecular characteristics of SFG (with its 2 subgroups) and control group

Variable	Stone formers group *N*:74			Control group *N*:40	P1	P2
	De novo *N*:40	Recurrent *N*:34	Total *N*:74			
Testosterone (2.62–16 ng/ml)						
Median (range)	7 (1.5–17.5)	4.5 (1–20)	5.3 (13.3–20)	7 (1.4–16)	0.8**	0.6**
Free testosterone (10-40 pg/ml)						
Mean ± SD	24 ± 11	23.5 ± 11	24 ± 11	24.7 ± 7	0.7*	0.8*
DHT (52–90 ng/dl)						
Mean ± SD	40 ± 15	41 ± 15	40.6 ± 15	37.5 ± 10	0.2*	0.7*
SHBG (10–71 nmol/l)						
Mean ± SD	37 ± 9	39 ± 9	37.8 ± 9	37.7 ± 9	0.9*	0.5*
Estradiol (up to 56 pg/ml)						
Median (range)	65 (20–226)	73 (20–300)	69.5 (20–300)	34 (20–55)	< 0.001**	0.8**
RQ-AR						
Mean ± SD	3.8 ± 1.3	3.5 ± 1.4	3.7 ± 1.3	1 ± 8	< 0.001*	0.5*
RQ-csf-1						
Mean ± SD	0.5 ± 0.19	0.4 ± 0.19	0.4 ± 0.19	1 ± 0.1	< 0.001*	0.6*
Mirna 185						
Mean ± SD	1.8 ± 0.3	1.9 ± 0.4	1.8 ± 0.4	1 ± 0.07	< 0.001*	0.4*

*P1* Total stone formers group vs control group

*P2* De novo vs control group

*SHBG* sex hormone binding globulin, *DHT* dihydrotestosterone

*Independent sample *t* test

**Mann–Whitney *U* test

However, RQ-csf-1 was significantly lower in SFG than control group (0.4 ± 0.19 vs 1 ± 0.1, respectively) with *p* < 0.001, while serum androgens and SHBG levels did not show significant difference between 2 groups.

### The secondary outcome

Table 2 summarizes data of SFG with comparative analysis of its 2 subgroups. Both de novo and recurrent stone formers group were comparable regarding serum androgens, estradiol levels and molecular gene expression of RQ-AR, RQ-csf-1 and Mirna 185 with *p* > 0.05.

Table 3 summarizes 24-h urinary metabolites among the 2 subgroups of stone formers with only urinary citrate level that showed significant higher median concentration in de novo stone formers (< 0.001).

**Table 3:** Comparison of metabolic work up between de novo and recurrent stone formers group

Variable	De novo group *N*: 40	Recurrent group *N*: 34	*P* value
24 h urinary Ca			
Median (range)	327 (125–3511)	410 (123–710)	0.3**
24 h urinary uric acid			
Median (range)	240 (122–921)	254 (201–698)	0.2**
24 h urinary oxalate			
Median (range)	55 (22–80)	47 (41–84)	0.2**
24 h urinary citrate			
Median (range)	354 (30–651)	526 (236–652)	< 0.001**
Hypercalciuria			
NO (%)	37 (92.5)	32 (94.1)	0.7^#^
Hyperuricosuria			
NO (%)	2 (5)	0	0.2^#^
Hyperoxaluria			
NO (%)	31 (77.5)	22 (64.7)	0.2^#^
Hypocitraturia			
NO (%)	14 (35)	5 (15.2)	0.05^#^

Reference range; hypercalciuria; > 200 mg/day, hyperuricosuria; > 750 mg/day, hyperoxaluria; > 45 mg/day and hypocitraturia; < 320 mg/day

**Mann–Whitney *U* test

^#^Chi square test

## Discussion

Kidney stones prevalence had markedly increased over the past 2 decades. According to the latest report from the National Health and Nutrition Examination Survey (NHANES 2007–2010), the prevalence of kidney stones among American adults was 8.8%: 10.6% among men and 7.1% among women [13].

Renal stones have major economic and medical burdens due to cost of treatment, sick leaves from work, risk of renal impairment and renovascular hypertension [15]. Another major problem of stone diseases is the risk of recurrence, 50% of patient with kidney stones have chance of development another episode of stone disease over 7 years [16].

CaOx stones are formed through a complex and multifactorial process including underlying genetic and metabolic abnormalities, life style pattern, obesity and hot climatic environment [1]. Recently, a strong association was found between sex hormones and kidney stones. Testosterone and dihydrotestosterone hormones were found to be a potential promoting factor in occurrence of CaOx urolithiasis, while estrogen hormone was found to be a protective against urolithiasis [3, 14].

This role was supported by prevalence of urolithiasis. It has been observed that women show a bimodal distribution of stone disease, demonstrating a second peak in incidence in the sixth decade of life corresponding to the onset of menopause and a fall in estrogen levels [7]. In addition, hyperoxaluria was found to be significantly lower in castrated male rates compared to normal ones [17]. Polycystic ovary syndrome which is characterized by clinical and laboratory evidence of hyperandrogenism is a known risk factor in kidney stones formation [18]. In some animal studies, administration of testosterone increases urinary oxalate excretion and enhances the formation of calcium oxalate stones [19].

This promoting effect of testosterone in urolithiasis pathogenesis is mediated through different mechanisms including increase hepatic synthesis of glycolic acid oxidase (GAO) which results in hyperoxaluria [11]. In addition, testosterone was found to inhibit renal osteopontin expression and subsequently increase renal excretion of oxalate [10]. In serum, 95% of testosterone binds sex hormone binding globulin and only 1% to 2% is free testosterone [20]. Free testosterone diffuses into target cells, where it binds to AR, which has the pivotal role in androgen signaling.

ARs were found to be expressed in nuclei of normal distal renal tubular epithelial cells. Moreover, they were found to be upregulated in tubular epithelial cells of patients with hyperoxaluria which suggests the important role of androgen/AR axis enhancement in kidney stones formation [9].

The molecular mechanisms of action of ARs in pathogenesis of hyperoxaluria and CaOx stones formation were demonstrated in 2 animal trials on mice [12, 21].

First, on physiological basis, there are Types of intrarenal macrophages: M1 macrophages which induce kidney epithelial injury and inflammation and not phagocytose CaOx crystals and M 2 macrophages which promote phagocytosis of CaOx crystals.

In this trial, they found that CSF-1 is mainly secreted from renal tubular epithelium and play an important role in M2 macrophages proliferation and in extension suppression of hyperoxaluria.

However, they found that AR increase miRNA expression which inhibit CSF-1 resulting in M2 macrophages inhibition and promoting hyperoxaluria.

In the second trial [21], they found that AR could directly upregulate hepatic glycolate oxidase and kidney epithelial NADPH oxidase subunit p22-PHOX at the transcriptional level. This upregulation might then increase oxalate biosynthesis and oxidative stress that resulted in induction of kidney tubular injury. Targeting AR with the AR degradation enhancer dimethyl curcumin (ASC-J9) led to suppression of CaOx crystal formation. However, these results were demonstrated only in animal studies.

Only one trial assessed the levels of ARs in human patients with CaOx stones [9]. This study included 68 participants, 37 male patients with CaOx/CaPh stones and 31 healthy controls. ARs were detected by immunohistochemistry in nuclei of normal distal renal tubular epithelial cells. Moreover, they were found to be upregulated in tubular epithelial cells of patients with hyperoxaluria which suggests the important role of androgen/AR axis enhancement in kidney stones formation. However, this study has some limitations of being small sample size and the detailed mechanism of ARs could not yet be fully elucidated.

From clinical point of view, knowing that the risk of recurrence of stone formation is about 50% [16], there was a need for a trial looking after molecular and genetic differences and if they have a role in stone recurrence. This might have clinical impact on attempts to reduce stone recurrence.

To the best of our knowledge, this prospective trial is the first one to compare the difference in sex hormones between de novo and recurrent stone formers and, the first one to study the relation between AR and genetic expression of miRNA and CSF-1 in stone former patients. Moreover, it includes a sample size larger than any other previous study.

In the current study, AR and miRNA were significantly higher and CSF-1 was significantly lower in SFG compared to control group. These results go in parallel with the previous result of [12] animal studies. However, no difference between both groups was observed regarding blood androgen level. Interestingly plasma estradiol level was higher among SFG in the current study, yet this could be explained by the higher rate of conversion of testosterone to estradiol in the testosterone metabolic pathway. However, this higher level did not seem strong enough to exert stone inhibiting effect in males [13].

Nevertheless, de novo and recurrent stone formers were not different regarding all studied parameters. Further studies are warranted to look for the clinical utility of those findings.

In conclusion, CaOx renal stones was found to positively correlated with genetic expression of AR and miRNA and it inversely related with CSF-1. However, renal CaOx stones’ recurrence was not associated with changes in sex hormone levels nor its related molecular pathway.

## Data Availability

All data relevant to this publication is readily available.
